# A New Look on Financial Markets Co-Movement through Cooperative Dynamics in Many-Body Physics

**DOI:** 10.3390/e22090954

**Published:** 2020-08-29

**Authors:** María Nieves López-García, Miguel Angel Sánchez-Granero, Juan Evangelista Trinidad-Segovia, Antonio Manuel Puertas, Francisco Javier De las Nieves

**Affiliations:** 1 Department of Economics and Business, University of Almería, 04120 Almería, Spain; mlg252@ual.es; 2 Department of Mathematics, University of Almería, 04120 Almería, Spain; misanche@ual.es; 3 Department of Chemistry and Physics, University of Almería, 04120 Almería, Spain; apuertas@ual.es (A.M.P.); fjnieves@ual.es (F.J.D.l.N.)

**Keywords:** econophysics, collective motion, finance, stock market, capital assets pricing model

## Abstract

One of the main contributions of the Capital Assets Pricing Model (CAPM) to portfolio theory was to explain the correlation between assets through its relationship with the market index. According to this approach, the market index is expected to explain the co-movement between two different stocks to a great extent. In this paper, we try to verify this hypothesis using a sample of 3.000 stocks of the USA market (attending to liquidity, capitalization, and free float criteria) by using some functions inspired by cooperative dynamics in physical particle systems. We will show that all of the co-movement among the stocks is completely explained by the market, even without considering the market beta of the stocks.

## 1. Introduction

The Capital Asset Pricing Model (CAPM) by Sharpe [1] Lintner [2] and Black [3,4] is one of the most relevant models within financial economics. The contribution of the CAPM to finance was associating the return on a financial asset with its systematic risk, which is measured by the market parameter Beta.

The CAPM approach to the study of the return on financial assets is as follows:

(1) $$r_{i}-R_{f}=\alpha_{i}+\beta_{i}\left(R_{m}-R_{f}\right)+\epsilon_{it}$$

where,

$r_{i}$ is the return on the asset *i*,
$\alpha_{i}$ is the part of the return on the asset *i* that is due to aspects of the asset itself, such as profit, business, etc.
$R_{f}$ is the return of the risk-free asset,
$R_{m}$ is the market return (usually proxied through its main equity index or an all-shares value-weighted portfolio),
$\left(R_{m}-R_{f}\right)$ is known as the market risk premium, and
$\epsilon_{it}$ is a zero-mean residual.

In a sense, the CAPM is used to explain the differences in risk premiums for different assets in the market, which are caused by differences in the risk of asset returns. According to the CAPM, $\beta_{i}$ is the factor that measures the sensitivity of the return on asset *i* to market movements. Accordingly, if the risk premium of a particular asset needs to be predicted, it is only necessary to select a risk-free rate and the beta of this asset. This model has been widely studied not only in the financial literature, but also by practitioners. However, there is a permanent debate about the usefulness of the market model, since, over time, empirical work has emerged with contrary results about whether market beta is indeed a useful factor in the study of a portfolio’s performance. Blume [5], Black et al. [6], and Fama and Macbeth [7] performed the first tests over the CAPM and stated that indeed it provides a good prediction model. Conflicting results were obtained by King [8], who found the existence of variance due to industry effects and that the market effect accounts for only about 50 per-cent of the variance. Meyer [9] also found numerous unexplained components that represent some persistent significant source of interdependence among stock prices. Banz [10] found that the size effect of firms explained better than market beta the variation in average returns at a portfolio. Roll [11] found that market beta explains monthly stock price changes only in an average of 30%. However, testing other factors, such as industry or size, this author concluded that explanatory power by systematic economic factors is not different across industries. Bhandari [12] also found a positive relationship between leverage and average return. This author concluded that beta leaves out important information in order to explain more accurately the reality. Based on previous results of Stattman [13] and Rosemberg et al. [14], Fama and French [15] proposed an alternative model including three factors: the market beta, the size effect and the book to market ratio. According to their results, the market beta barely had the capacity to explain the variations in the average yields of the selected stocks, while size and value factors showed interesting results. The new factorial model proposed by Fama and French made clear the insufficiency of the market beta to cover all of the information needed to explain the variations in returns. Over time, advocates of the market beta have been studying the reasons why the CAPM no longer performs well in empirical studies. A good example is found in Isakov [16]. In this paper, the authors stated that beta has no chance of being a useful variable in recent empirical studies for two reasons. The first reason is that the model is expressed in terms of expected yields, but the tests can only be performed on yields that have been realized. The second reason is that excess realized market returns do not behave as expected, i.e., they are too volatile and often negative. There are similar works with different methodologies proposed, where it is shown that the market beta is still valid under some circumstances (see, for example, Pettengill, Sundaram, and Mathur [17], Chan and Lakonishok [18], Grundy and Malkiel [19], or Lopez et al. [20]).

For our purposes, one of the main goals of the CAPM is to explain the correlation of different assets through its relationship with the market index given by Equation (1). This represented an important advance in portfolio selection, because the market index is expected to explain all of the co-movement between two different assets, leaving only the residual $\epsilon_{it}$ to differ the trend of asset prices.

Assets co-movement has captured the attention of researchers due to its importance for asset allocation, portfolio diversification, or risk management. Causes of co-movement have been studied from different points of view. Roll [11] found that the level of stocks co-movement depends on the relative amounts of firm-level and market-level information capitalized into stock prices. Domowitz et al. [21] proved that liquidity co-movement is determined by order flow and return co-movement is caused by order type (market and limit orders). Byrne et al. [22] found that co-movement is responsible for two-thirds of variability in global bond yields. These authors concluded that global inflation explains most of the global yield co-movement.

Morck et al. [23] found that stock returns are more synchronous in emerging markets. The authors also show evidence that co-movement is not a consequence of structural characteristics of economies, such as fundamentals volatility or country and market size. Jach [24] quantified time-varying, bivariate, and multivariate co-movement between international stock market return. This work concluded that development and region are not always decisive factors. Parsley and Popper [25] observed that return co-movement does not depend on the richness of the country, but it is more affected by variables that reflect different institutional aspects, including international macroeconomic policy stability.

Other authors have focused their attention on the co-movement between different assets or countries. Bonfiglioli and Favero [26] did not find evidence of long-term interdependence between US and German stock markets. Rua and Nunes [27] analyzed the co-movement across major developed countries among 40 years. These authors found that the co-movement is strong across countries in lower frequencies and it is different in each one. They also remarked that the degree of co-movement varies over time. Akoum et al. [28] examined the co-movements of stock markets in the GCC region and crude oil prices. These authors showed evidence of a strong dependence after 2007 in the long term. However, in the short term, there is no clear evidence of dependence. Reboredo [29] found no extreme co-movement between oil prices and exchange rates in the periods before and after the financial crisis. Magdaleno and Pinho [30] reported a strong and significant relationship between index prices. The authors show that innovations in the US and UK stock markets are not rapidly transmitted to other markets. They also found that, economically, as well as geographically, economies show higher levels of co-movements, except Japan. Loh [31] studied the co-movement of 13 Asia-Pacific stock markets and the European and US ones. These authors found a significant co-movement between most of them in the long run. During the financial crisis, this author reports evidence of important variation in co-movement in time.

Factors revelant in co-movement have also been analyzed by some authors. Baca et al. [32] showed evidence of the importance of global industry factors in explaining international return variation. In a similar line, Cavaglia et al. [33] showed that country effect is more relevant than industry factors in the late-1990s and Griffin and Karolyi [34] found that global industry factors only explain four percent of the variation in local stock markets. However, L’Her et al. [35] reported evidence in an opposite sense, finding that global industry effects surpassed country effects in importance in 1999–2000. Brooks and Del Negro [36] analyzed the link between international stock market co-movement and firm level variables that measure international diversification, finding a significant relationship between stock returns betas and those variables. Antonakakis and Chatziantoniou [37] showed that there is a clear negative correlation between the policy uncertainty and stock market returns, except during the financial crisis.

Different approaches have been used to measure co-movement, such us cross-correlation analysis (Akoum et al. [28]), spatial techniques (Fernández-Aviles et al. [38]), regression coefficient (Brooks and De Negro [36]), quantiles (Cappiello et al. [39]), tail-dependence coefficient (Garcia and Tsafack [40]), copula approach (Rebodero [29]), time series analysis (Antonakakis et al. [37]), shortfall-multidimensional scaling approach (Fernández-Aviles et al. [41]), or a recent approach based in Hurst Exponent (Ramos Requena et al. [42]). In this paper, we propose a new approach to study the co-movement of the whole market based on some functions borrowed from or based on physical particle systems, following our previous works on this topic (Clara Rahola et al. [43], Sánchez Granero et al. [44], Puertas et al. [45]).

From a physical point of view, a portfolio can be seen as a many-body system, with the index representing a particular point of the system characterizing the whole system, such as the center of mass. The main contribution to the asset motion is described by Brownian motion, and the interactions among the assets are unknown, if they exist, and they induce collective motion. However, as given by classical mechanics of many-body systems, internal forces only affect the relative motion of a body with respect to the center of mass. Thus, a proper account of cooperative motion, within a physical approach can only be made if the center of mass (or index) is subtracted.

We study co-movement in the whole market in this paper by considering some functions inspired by co-movement in physical particle systems. It is showed that the market (represented by an equal weighted portfolio of all stocks) explains all of the co-movement in the whole market. Alternative ways to represent the market are considered, for example, portfolios weighted by capitalization or known indexes, in order to check whether these representations are also able to explain all the co-movement in the market. Some other alternatives that take into account the market beta are also considered.

## 2. Methodology

Cooperative dynamics have been observed in many physical particle systems. Furthermore, due to the short-time Brownian motion of assets, here we focus on particles with Brownian dynamics, namely colloidal systems. In colloids, as well as in atomic or molecular fluids, upon lowering the temperature the dynamics of the system slows down until the glass transition is reached, and the dynamics freezes, ideally. Accompanying this slowing down, the dynamics becomes more cooperative with more mobile particles clustering together in string-like structures (Donati et al. [46], Cates and Evans [47], Weeks et al. [48]). To study this phenomenology, several so-called four points correlation functions have been proposed, relating the dynamics of two particles at two different times (Glotzer et al. [49], Berthier et al. [50]). The most direct one is the evolution of the distance between a given pair of particles (Muranaka et al. [51], Zahn et al. [52]). Inspired by these functions, here we propose three simple observables that can be used to monitor the cooperative dynamics in financial systems:

- $C_{1}$. Consider the functions
  $$C_{1t}\left(\tau \right)=\frac{\sum_{i,j}{\left(\delta x_{i}\left(t,\tau \right)-\delta x_{j}\left(t,\tau \right)\right)}^{2}}{\sum_{i,j}\left(\delta x_{i}{\left(t,\tau \right)}^{2}+\delta x_{j}{\left(t,\tau \right)}^{2}\right)}$$
  $$C_{1}\left(\tau \right)=<C_{1t}\left(\tau \right)>$$
  where $x_{i}\left(t\right)$ is the log-price of asset *i* at time *t*, $\delta x_{i}\left(t,\tau \right)=x_{i}\left(t+\tau \right)-x_{i}\left(t\right)$ and the sum is for each pair of assets *i*, *j*, excluding the pairs *i*, *i*. $C_{1}$ is the average of $C_{1t}\left(\tau \right)$ over time origins *t*.
  The function $C_{1t}$ is a measure of co-movement, from time *t* to time t+τ, along the time *t* and the function $C_{1}$ is a measure of co-movement for the full period considered.
  The interpretation of these functions is as follows: if the resulting function is close to 1, it means that there is no co-movement in the whole market; if it yields values lower than 1, then we can say that the stocks move together; and if, on the contrary, the values are greater than 1, the stocks tend to move in the opposite direction.
- $C_{2}$. Consider the functions
  $$C_{2t}\left(\tau \right)=\frac{\sum_{i,j}\delta x_{i}\left(t,\tau \right)\delta x_{j}\left(t,\tau \right)}{\sum_{i,j}\left(\delta x_{i}{\left(t,\tau \right)}^{2}+\delta x_{j}{\left(t,\tau \right)}^{2}\right)}$$
  $$C_{2}\left(\tau \right)=<C_{2t}\left(\tau \right)>$$
  where the same notation is used.
  In this case, if the functions are close to 0 it means that there is no co-movement; if they are greater than 0, it means that the stocks move in the same direction and, when they are less than 0, the stocks move in the opposite direction.
- $C_{3}$. Consider the functions
  $$C_{3t}\left(\tau \right)=\frac{\sum_{i,j}\delta x_{i}\left(t,\tau \right)\delta x_{j}\left(t,\tau \right)}{\sum_{i,j}\left|\delta x_{i}\left(t,\tau \right)\delta x_{j}\left(t,\tau \right)\right|}$$
  $$C_{3}\left(\tau \right)=<C_{3t}\left(\tau \right)>$$
  where the same notation is used.
  These functions are interpreted in the same way that $C_{2}$. Note that $C_{3t}$ and $C_{3}$ take values between −1 and 1.

Note that functions $C_{1}$ and $C_{2}$ are analogous to functions $\gamma \left(\tau \right)/<{\left(\delta x\right)}^{2}>$ and C(τ) in Puertas et al. [45], respectively, while function $C_{3}$ is a variation of $C_{2}$. The three functions are intended to measure the degree of co-movement among all the stocks in the market, though they are slightly different.

## 3. Results

In this work, more than three thousand stocks (attending to liquidity, capitalization and free float criteria) in the USA market are considered from 2003 to March 2020. We will first show the results of the three functions proposed above, and the importance of subtracting the market to the evolution of the asset prices. Next, we will show how these parameters evolve in time and can be used to identify high-volatility periods. In the final subsection, other forms to represent the market are studied.

### 3.1. Co-Movement for Different Lag Times with and without Considering the Market

In this section, we want to study the behavior of the USA market through functions that show the co-movement of the shares. To calculate the shares, we will use, as data, the logarithm of the share price in two specific years, 2008 and 2018, in order to be able to check how the co-movement is affected in crisis stages and other more stable ones.

We will calculate the functions of the co-movement in two different ways: the first as described in the previous section, and the second by subtracting the market average, which is, $\delta x_{i}\left(t,\tau \right)=x_{i}\left(t+\tau \right)-x_{i}\left(t\right)-\left(m\left(t+\tau \right)-m\left(t\right)\right)$, where $x_{i}$ is the log price of asset *i* and $m=<x_{i}>$ is the market (the mean of the log-price of all assets). The purpose is to check how the market average affects the stock co-movement calculations. Note that $m\left(t+\tau \right)-m\left(t\right)=<x_{i}\left(t+\tau \right)-x_{i}\left(t\right)>$, which is, the market log-return is the equally weighted mean of the log-return of all the stocks.

The subtraction of the market is equivalent to removing the motion of the center of mass in physical systems. There, the dynamics of a system of particles can be separated in the motion of the center of mass, and the dynamics respect to it. Whereas external forces affect the center of mass, the internal dynamics is controlled by the internal forces.

We will use the three functions described in the previous section to measure the degree of co-movement.

**Figure 1:**
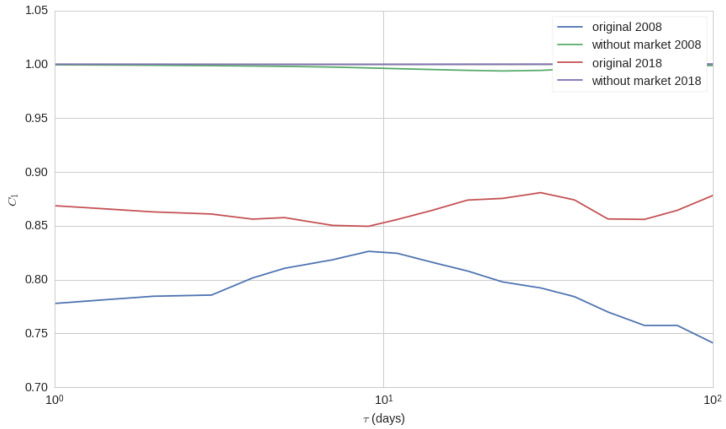
Co-movement with $C_{1}$ function. A value of 1 means that there is no co-movement, while values of less than 1 mean that the stocks tend to move in the same direction.

**Figure 2:**
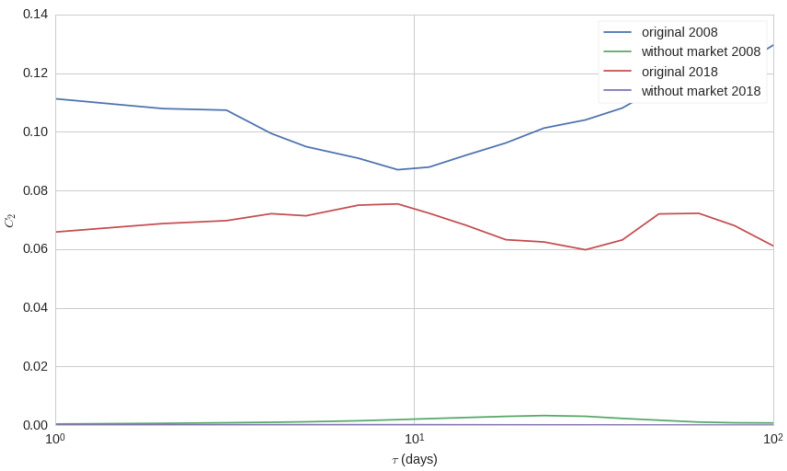
Co-movement with $C_{2}$ function. A value of 0 means that there is no co-movement, while values greater than 0 mean that the stocks tend to move in the same direction.

**Figure 3:**
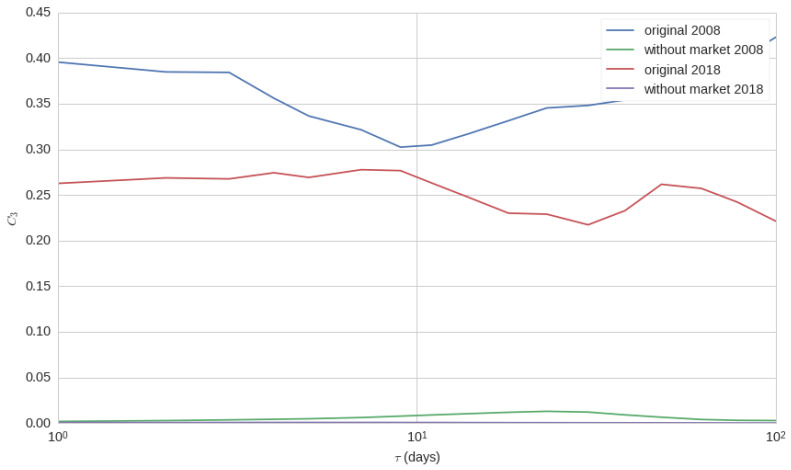
Co-movement with $C_{3}$ function. A value of 0 means that there is no co-movement, while values that are greater than 0 mean that the stocks tend to move in the same direction.

In Figure 1, Figure 2 and Figure 3, we show the results that were obtained with $C_{1}$, $C_{2}$, and $C_{3}$, respectively, for the years 2008 and 2018, depending on whether or not the market average is taken into account.

In both years and for the three functions, it is clear that there is some co-movement in the whole market (in the three cases, the co-movement in the year 2008 is a bit greater than in the year 2018), but all of the co-movement is explained by the market, since the co-movement disappears when we subtract the market.

Because the three functions essentially provide the same information, we use in the following $C_{3t}\left(\tau \right)$ and $C_{3}\left(\tau \right)$.

### 3.2. Co-Movement along the Time

To study the evolution of the co-movement with a wide time horizon, we have used the function $C_{3t}\left(20\right)$, which is, the degree of co-movement after 20 working days.

**Figure 4:**
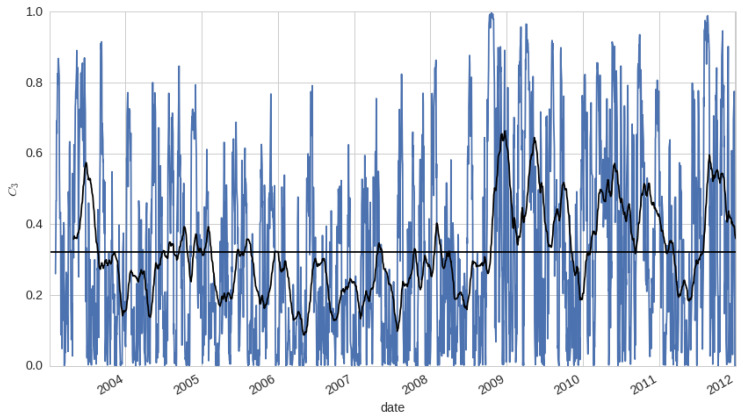
Co-movement ($C_{3t}\left(20\right)$) of the whole market from 2003 to 2011. Blue line is the daily co-movement, while black line is a moving average of the blue line with a time window of 60 trading days.

**Figure 5:**
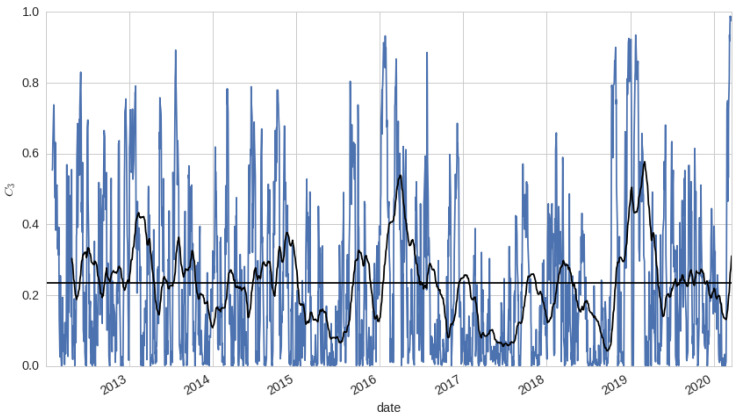
Co-movement ($C_{3t}\left(20\right)$) of the whole market from 2012 to 2020. Blue line is the daily co-movement, while black line is a moving average of the blue line with a time window of 60 trading days.

In Figure 4 and Figure 5, we show the function $C_{3t}\left(20\right)$ for the period (2003–2020). A moderate degree of co-movement can be observed at all times. Note that the co-movement is higher during crisis moments or bear markets: 2003 with the end of the dot.com bubble, the end of 2008 and the beginning of 2009 with the financial crisis, mid 2010 with the flash crash, mid 2011 with the Eurozone debt crisis, mid 2015 to early 2016 with the Chinese stock market crash, the end of 2018 with the close to 20% decline on major indexes and mid 2020 with the coronavirus crisis. At some particular days, the co-movement is very close to 1, which is an astonishing value of co-movement. Similar results have been reported by Tseng and Li [53] and Trinidad et al. [54].

**Figure 6:**
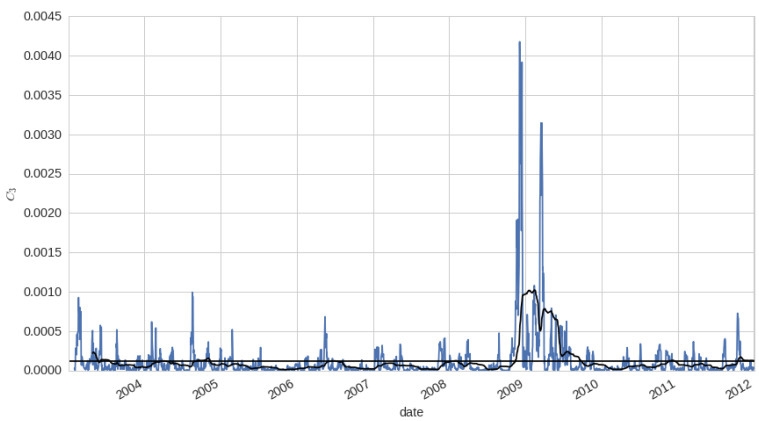
Co-movement ($C_{3t}\left(20\right)$) of the whole market from 2003 to 2011 with the market removed. Blue line is the daily co-movement, while black line is a moving average of the blue line with a time window of 60 trading days.

**Figure 7:**
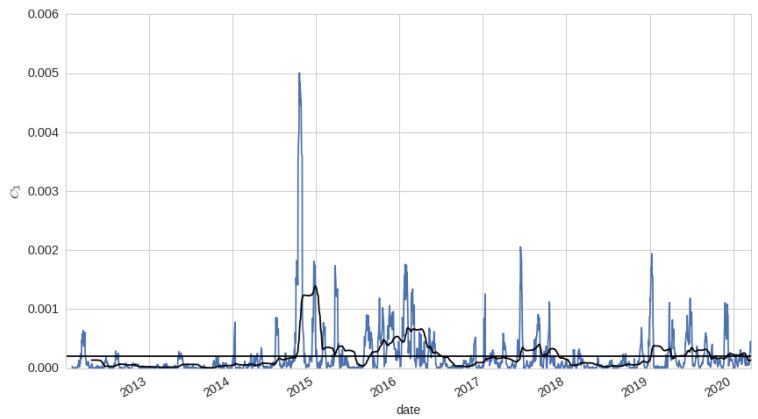
Co-movement ($C_{3t}\left(20\right)$) of the whole market from 2012 to 2020 with the market removed. Blue line is the daily co-movement, while black line is a moving average of the blue line with a time window of 60 trading days.

In Figure 6 and Figure 7, we show $C_{3t}\left(20\right)$ with the market subtracted, as explained in the previous section. Clearly, no co-movement is detected with this observable. The highest punctual peaks are less than 0.005.

We can conclude that the market completely explains the co-movement among the stocks, which is quite a surprising result, since we have not considered any beta relationship.

### 3.3. Other Ways to Represent the Market

In this section, we have made the calculations taking into account different ways of representing the market, to see in which scenarios the co-movement is more reduced. Therefore, $\delta x_{i}\left(t,\tau \right)$ is calculated as $\delta x_{i}\left(t,\tau \right)=x_{i}\left(t+\tau \right)-x_{i}\left(t\right)-\left(m\left(t+\tau \right)-m\left(t\right)\right)$, where $x_{i}$ is the log price of asset *i* and m(t) represent the market. The ways of studying the market that we have selected are:

- ew: equal weight. This is the representation described in the previous section, which is, $m\left(t\right)=\frac{1}{N(t)}\sum_{i}x_{i}\left(t\right)$, where N(t) is the number of stocks at time *t*.
- cap: this representation is calculated as a capitalization-weighted average, which is, $m=\sum_{j}w_{j}x_{j}$ and $w_{j}=c_{j}/\sum_{k}c_{k}$ with $c_{j}$ the capitalization of asset *j*.
- SPY: the SP500 index.
- IWM: the Russell 2000 index.

**Scheme 1:**
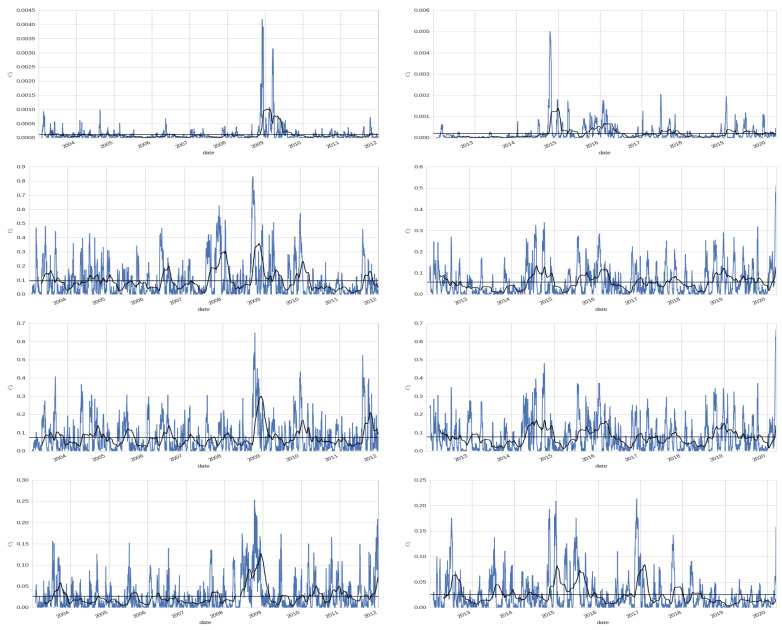
Co-movement ($C_{3t}\left(20\right)$) of the whole market during 2003–2011 (left) and 2012–2020 (right) with the market removed. Blue line is the daily co-movement, while black line is a moving average of the blue line with a time window of 60 trading days. The representation of the market is, from top to bottom: ew, cap, SPY, IWM. The horizontal line represent the average co-movement ($C_{3}\left(20\right)$) along the full period.

In the previous section, we studied the co-movement in two periods 2003–2011 and 2012–2020, and found that, if we measure the market with the ew method, the co-movement in these two periods is very close to zero. In Scheme 1, it is shown what happens when we consider other ways to represent the market. It can be seen that the representations of the market that explain the co-movement better than any of the alternatives considered are ew.

#### Considering Beta

As we commented in the introduction, the market beta is used in the literature to model the log-return of a stock with respect to the log-return of the market. The simplest model is the Sharpe one, but other models, like the Fama–French factor model, also consider the market beta as a part of the model. Subsequently, the log-return of a stock is modeled as $x_{i}\left(t+\tau \right)-x_{i}\left(t\right)=\alpha_{i}+\beta_{i}\left(m\left(t+\tau \right)-m\left(t\right)\right)+\varepsilon_{i}$, where $\varepsilon_{i}$ follows a fixed stationary distribution with zero mean and is independent of the market. It follows that $\frac{x_{i}\left(t+\tau \right)-x_{i}\left(t\right)}{\beta_{i}}-\left(m\left(t+\tau \right)-m\left(t\right)\right)=\frac{\alpha_{i}}{\beta_{i}}+\frac{\varepsilon_{i}}{\beta_{i}}$, and hence it is independent of the market.

To check this model, in this case, $\delta x_{i}\left(t,\tau \right)$ is calculated as $\delta x_{i}\left(t,\tau \right)=\frac{x_{i}\left(t+\tau \right)-x_{i}\left(t\right)}{\beta_{i}}-\left(m\left(t+\tau \right)-m\left(t\right)\right)$, and *m* is represented in the different ways described previously (ew, cap, SPY, IWM). Assuming the beta model, the co-movement that is calculated in this way should be lower than the co-movement calculated without considering the beta.

**Scheme 2:**
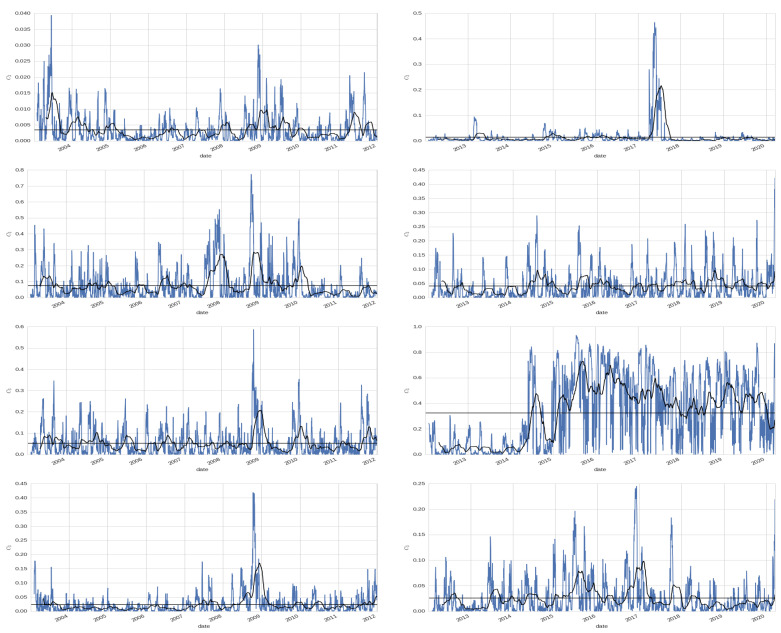
Co-movement ($C_{3t}\left(20\right)$) of the whole market during 2003–2011 (left) and 2012–2020 (right) with the market removed when considering the beta of each stock. The blue line is the daily co-movement, while black line is a moving average of the blue line with a time window of 60 trading days. The representation of the market is, from top to bottom: ew, cap, SPY, IWM. The horizontal line represent the average co-movement ($C_{3}\left(20\right)$) along the full period.

In Scheme 2, we show the co-movement ($C_{3t}\left(20\right)$) calculated within the Fama and French model. We can say that this version does not improve the results obtained previously and hence the previous model is preferred, since the results are better and the model is simpler.

Therefore, from the point of view of co-movement, we can conclude that the relationship between the log-returns of a stock and the market is provided by $x_{i}\left(t+\tau \right)-x_{i}\left(t\right)=\alpha_{i}+\left(m\left(t+\tau \right)-m\left(t\right)\right)+\varepsilon_{i}$, which implies β=1.

## 4. Conclusions

In this paper, a new approach to study co-movement among the stocks of the whole (USA) market is considered, by using some functions that are inspired by cooperative dynamics in physical particle systems. We prove that these functions identify the economic crisis as periods of increased co-movement, which is agreement with the finding of previous research (see, for example, Tseng and Li [53] and Trinidad et al. [54]). From this perspective, the market (represented by an equally weighted portfolio of all the stocks considered) completely explains all of the co-movement among the stocks in such a way that, if we remove the market, the co-movement is almost zero. This fact supposes an important finding considering that one of the most important inconveniences of beta estimations is their instability in time.

The three functions are three different ways to measure the whole co-movement of the market. The fact that the results are similar implies that the measure is quite robust. Therefore, these measures seem to be quite useful, since they provide a way to measure the co-movement among the stocks and we can obtain results that are not obvious. Moreover, we think that these measures open a new door to study the co-movement among a set of stocks for different purposes.

Other ways to represent the market (capitalization weighted, SP500 index and Russell 2000 index) can also explain the co-movement in the market, but to a lesser extent than an equally weighted portfolio. This result is similar to a recent finding of Lopez et al. [20], where the authors show that a market factor represented by an equally weighted portfolio is highly significant in a factor model.

To conclude, the market is also considered taking into account the market beta of the stocks, but again, the results are not as good as the simpler equally weighted portfolio. These results lead us to propose a modification of the CAPM model, removing the beta in the classic model.
